# Medical students’ experience and learning outcomes of overseas community involvement project: a qualitative study

**DOI:** 10.1186/s12909-024-05560-6

**Published:** 2024-08-08

**Authors:** Gayathri Devi Nadarajan, Kumaran Rasappan, Jonathan Shen You Ng, Melvin Lim Junchen, Sungwon Yoon

**Affiliations:** 1 Department of Emergency Medicine, Singapore General Hospital, Singapore, Singapore; 2 SingHealth Duke Global Health Institute, Singapore, Singapore; 3 Department of Orthopedic Surgery, National University Health Systems, Singapore, Singapore; 4 Ministry of Health Holdings, Singapore, Singapore; 5 Health Services and Systems Research, Duke-NUS Medical School, Singapore, Singapore

**Keywords:** Medical mission, Medical volunteerism, Humanitarian medicine, Experiential learning, Reflective learning, Transformative learning

## Abstract

**Background:**

Medical students in Singapore engage in short term medical missions, locally known as Overseas Community Involvement Projects (OCIPs). Little is known about the learning outcomes of an OCIP and how this complements their medical education back home. Understanding this can help the medical educators structure the OCIP to optimise its learning value.

**Objectives:**

This study aims to gain an in-depth understanding of the experiences and learning outcomes of the medical students who participated in the OCIP.

**Methods:**

This was a qualitative study involving Singaporean students from one medical school travelling to Nepal. Data was collected from reflective journals, overall group reflections and two focus group discussions. The data was thematically analysed using the Accreditation Council for Graduate Medical (ACGME) core competencies for medical professionals.

**Results:**

The data could be classified under various themes within the six domains of the ACGME framework. The study revealed themes of: humanism, socioeconomic and cultural determinants of health under the domain of patient care, application of medical knowledge, investigating and evaluating the needs of a population and feedback to drive improvement under the domain of practice-based learning and improvement, use of non-verbal cues and communicating across language barriers under the domain of interpersonal and communication skills, healthcare systems and delivery, resourcefulness and adaptability, health equity and accessibility under the domain of systems-based practice, ethics, role-modelling, teamwork and leadership skills, interprofessional skills and resilience under the domain of professionalism. Understanding the students’ motivations, utilising reflections, and following the patients’ journey facilitated attainment of these outcomes.

**Conclusions:**

This OCIP experience translated to learning outcomes aligned with the ACGME framework. There is great potential for the experiential learning from a well-structured OCIP to help with personal and professional development and global health education.

**Supplementary Information:**

The online version contains supplementary material available at 10.1186/s12909-024-05560-6.

## Introduction

Globalisation provides opportunities and challenges to medical education. There is an increasing interest in overseas service trips and global health education where medical students engage in Overseas Community Involvement Project (OCIP). These trips involve medical students, usually from high income regions, travelling to a lower resource setting. The trips, ranging from 1 week to 3 weeks in duration, are student-led, supervised by a physician mentor who may not necessarily accompany them. Such trips were more common prior to the COVID-19 pandemic related travel restrictions. Currently, it is picking up pace once again as the world is steadily recovering from the pandemic.

As this overseas service activity begins to resume, it is time to re-think how it can be approached. These trips raise ethical issues such as sustainability of student involvement or a lack of follow-up of patients after a diagnosis of a chronic illness during the trip [1–4]. In addition, when students volunteer, significant resources are dedicated to this activity including time, money and even utilisation of the receiving countries’ scarce resources. Safety of all involved is also an issue as there are no regulations about personal protective equipment or operating protocols should the students encounter an infectious disease outbreak. Hence, it is now more important than ever to be clear on the risks and benefits of such trips.

If the risks and benefits are well taken into consideration when planning an OCIP, these trips may have the potential to benefit the community in low-income settings through collaborative partnerships [5, 6]. For the students, the OCIP may serve as an educational tool or pedagogy in medical education. The experience can be very rich and may stimulate learning of important but often neglected topics within medical education which are also challenging to teach such as health systems and socioeconomic and cultural determinants of health [7–9]. The OCIP also provides students with early exposure to community health [10] and may potentially be a valuable source of experiential learning.

While a few studies [11–13] described the benefits and issues around volunteerisms in global health, very little medical education research has been conducted to demonstrate the possible learning outcomes of an OCIP. Specifically, there is a gap in understanding how the OCIP experience relates to medical education competencies. Furthermore, the literature on the experience of Asian medical students volunteering in overseas community projects is sparse. As global health issues are increasingly incorporated into medical education with growing interest in OCIP, it is important to understand what Asian medical students learn and how this complements their medical education back home. This in turn can help the medical educators structure the OCIP to optimise its learning value. Therefore, the aim of this study is to gain an in-depth understanding of the experiences and learning outcomes of the medical students who participated in the OCIP.

## Methods

### Study design

A qualitative study design was chosen as the study requires an in-depth understanding of students’ experiences.

### Setting and participants

This study was based in one medical school in Singapore, a metropolitan city state. Each of the three medical schools in Singapore have multiple OCIPs which are student-led with physician oversight. Such OCIPs have been in existence in each of these schools since their setup and is voluntary. It is currently not part of the medical curriculum but is available for anyone to join in medical school. The term OCIP is used rather than short term experiences in global health (STEGH) because the objective of such trips is to provide service to an underserved community rather than a ‘global health experience’. The OCIP group usually revisits the same location to ensure continuity of care. The OCIP activities typically include screening camps, health education or training to equip the community with a certain set of skills. Project Aasha is an annual OCIP where participants spend two weeks in the rural, mountainous region of Nepal. Landlocked between India and China in Asia, Nepal has a population of about 30 million, spread across the valley of Kathmandu (its capital) and unique terrains comprising of the world’s highest mountains and terai (lowland region). Though healthcare is heavily subsidised for the poor, the challenging terrains affects accessibility, and the poor health literacy and volatile politics makes implementation of policies difficult. Hence, universal health coverage and equitable health provision is still a struggle. The trip was based in Bung village in the Himalaya mountains in North-eastern Nepal at an elevation of 1800 m and Biratnagar city, a terai in Eastern Nepal. The health service consisted of first aid training and women’s health education for school students, health screening and cataract surgery for the villagers. This trip rooted from the local community leaders approaching the physician mentor of Project Aasha. They were concerned of the general poor state of health of the villagers- where many of them do not continue with follow up care for their chronic conditions, there was poor health literacy and there was a major concern about injuries and the lack of first aid knowledge as the nearest hospital was a day’s walk away. Following contact with the community leader, and prior to this trip, Project Aasha members did a separate trip for a needs analysis (by performing a door-to-door survey) and also proceeded to apply for permits to allow the team to practice in the village of Bung.

The OCIP team consisted of a physiotherapy group (four students and a mentor) an ultrasonographer, five doctors (from specialties of Ophthalmology, Emergency Medicine, Orthopaedics and Surgery), fourteen medical students and two Nepalese student translators. Pre-trip, the students were involved in researching about the community they would be visiting in Nepal, preparing the logistics for the team’s stay, trip itinerary, medical equipment, medications and training materials for the community. There was also a sharing session in which the teams that had previously went to Nepal shared their experiences. Once in Nepal, the team reached their destination by jeep on partially built gravel roads and trekking through the mountainous terrain. The team took the same route that the villagers would take to reach secondary and tertiary healthcare facilities. This gave them the opportunity to meet the various stakeholders in the clinics and hospitals, with the aim of understanding the local health system. The mentors accompanying the students utilised reflections [14] to consolidate students’ daily experiences. Reflective learning, where a deliberate attempt is made to share and reflect on one’s experiences from the day [15] is key to Project Aasha as it helps shape the experiences into learning moments. Participants for this study were 14 medical students who took part in the trip.

### Data collection

At the start, the students were asked to share verbally within the group and in a reflective log on their motivations to participate in the OCIP. On each of the four service days, they were also asked to fill a personal daily reflective log. The end of each service day consisted of a daily debrief, where operational issues of the day will be discussed followed by a group reflective session, where the supervising seniors (doctors and physiotherapists in this trip) also shared their reflections. The reflective log asked all the students to pen down their experience and learning points for the day while the group reflective session asked some of them to share their experiences from the day. This was recorded and transcribed verbatim. At the end of the trip, two focus group discussions (FGD) were conducted on-site where the students were asked to share their overall experience from the trip and what they have learnt. They were audio-recorded and transcribed verbatim. Hence, the data collection comprised three different sources- reflective journals, overall group reflections and the two FGDs.

### Data analysis

The transcribed data and written materials were thematically analysed by two coders (GN, MN). GN and MN are both medical doctors with public health training. Disagreements were resolved by a third coder (SY) who is an academic faculty member with expertise in global health and health services research through iterative meetings. Following the initial thematic analysis, compiled themes and sub-themes were subsequently mapped onto the ‘Accreditation Council for Graduate Medical (ACGME) core competencies for medical professionals’ since it is a commonly used framework to measure the competencies of the medical doctors in Singapore. Themes and sub-themes that did not fall within the ACGME categories but emerged from data were also compiled. Therefore, our analysis involved both inductive and deductive approaches. To bolster the strength of our qualitative analysis, we employed data triangulation by incorporating multiple data sources including on-site reflective journals, recorded group reflections and focus groups. These sources allowed us to capture comprehensive exploration of students’ experiences. The analysis involved two independent coders, each responsible for examining the three sources of data. By comparing interpretations of the coding, we assessed the extent of convergence across various data and between coders while also identifying any divergences. This approach ensured a rigorous examination of the experiences and learning outcomes. Through analysis, a conceptual diagram for the learning outcomes from OCIP was generated.

### Ethics

The study was declared to have exempt status and ethical waiver by the SingHealth Centralised Institutional Review Board (Ref no. 2018/3226).

## Results

Table 1 shows the characteristics of participants and their motivations to join the trip. There was a balanced number of male and female participants from both year one and two of the same medical school, with an average age of 20 years old. Approximately three quarters (75%) did not have a prior OCIP experience. The majority of students (50%) stated that the experience of healthcare in a low resource setting was the main motivation to join the trip, followed by the experience of healthcare within a different culture, learning how to plan for medical mission trips, wanting to join a sustainable OCIP project and an interest in serving an underserved population.

**Table 1:** Participant characteristics

	*N*. (%)
Gender	
Male	8 (57)
Female	6 (43)
Nationality	
Singaporean	12 (86)
Non-Singaporean	2 (14)
No. of previous trips	
0	6 (43)
1	7 (50)
2	1 (7)
Medical school year	
Year 1	7 (50)
Year 2	7 (50)
Average Age (SD)	20 (± 0.9)
Reasons for joining the trip	
Experience of healthcare & management of patients in a lower resource setting	7 (50)
Experience of healthcare within a different culture	2 (14)
Learn how to plan for medical mission trips	2 (14)
Sustainable nature of the OCIP	4 (28)
Interest in serving	3 (21)

### Learning points during the preparation phase of the trip

Table 2 shows what participants learnt during the pre-trip preparation phase. Three themes were identified – organisational skills, teaching skills and the ability to take into context the culture of the recipient community when developing health education materials. As this is a student-led trip, the students organised all aspects of the trip for the team as well as for the Nepalese community. Consequently, many reflected on acquisition of organisational skills during the preparation phase.

**Table 2:** Learning points during the preparation phase of the trip

Theme	Illustrative quotes
Organisational skills	• “Preparing for such OCIPs can be very logistically heavy and requires detailed and early planning. It is also important to delegate the responsibilities well for a large-scale project like this.” • “I have learnt to liaise with various parties in order to achieve a common goal…” • “I have learnt that communication plays a very important role.” • “I’ve learnt the importance of being meticulous.”
Teaching skills	• “I have gained a better understanding in how to teach even across language barriers, by the use of more demonstrations and hands-on…” • “Furthermore, it is very important to put ourselves in the shoes of the Nepali locals to understand their needs and wants to better target our health camps and education for better effectiveness.”
Cultural context in health education	• “Though not much is known yet about the actual situation in Bung, we conducted preliminary secondary research about the situation of menstrual health in Nepal. To most of us, hygiene and sanitation might be straightforward and easy to maintain. However, it may not be the case in Nepal due to certain traditions, beliefs and myths in their culture that may have influenced the way they feel about menstruation.”

The students also prepared teaching materials for first aid, hand hygiene and women’s health under the physician’s guidance. The initial teaching materials were adopted from the internet, which lacked localisation and thus appeared to be unsuitable for the villagers. For example, for menstrual hygiene, the menstrual cup was seen to be too invasive and culturally inappropriate, and the sanitary napkins were viewed as environmentally unfriendly as compared to using a cloth. Through feedback from the physicians as well as sharing from their predecessors who had visited the village the year before, they learnt to tailor the teaching materials accordingly to the local culture, beliefs and practices.

### OCIP experience and learning outcomes according to ACGME framework

Participants’ experiences and reflections engendered various themes under the six domains of the ACGME framework. These quotes for the themes are summarised in Table 3.

**Table 3:** OCIP experience and learning outcomes mapped onto ACGME framework

Domain	Themes	Illustrative quotes	No. of relevant responses
Patient care (PC)	Humanism	“She did something that perhaps none on the trip did which is to spend time to listen a patient extensively…I was more focused on getting a good spirometer result that helping the patient get better. Hopefully, I will remember this lesson for time to come, to open my mind and listen to patients.” “The patients should always be placed on the highest priority and doctors should not lose sight of the human side of medicine.”	10
	Socioeconomic determinants of health	“At night, K shared about an elderly couple with social issues that greatly outweighed their medical ones, leading me to rethink how we treat patients. The medical issues they face are probably often only a small area of what affects them, and in saying so, it is up to us to build a rapport with the patient and be able to discern what the patient is facing and needs.” “Often a times, social factors such as financial difficulties, inconvenience and language etc are the ones which limit the treatment of patients…Hence, social factors have to be considered very carefully in large scale policy planning and also in smaller scale dealing with patients and getting to understand them better so as to propose the best possible treatment plan.”	13
	Cultural determinants of health	“Since menstruation and menstrual health practices are closely linked to the culture and beliefs of the society, it is not right to condemn their practices or beliefs outrightly even if it doesn’t align with what we are used to.” “I learnt that culture and personal beliefs are sensitive things that should be treated carefully as most of these people have had these beliefs majority of their life. If we were to try to educate them on some aspects of their practices that we think can be improved, we would have to be sensitive and polite about it.”	11
Medical knowledge (MK)	Application of medical knowledge	“While examining patients, we used our medical knowledge to correlate the clinical presentation with the disease.” “Today also provided me with exposure to some clinical cases and helped me sharpen my clinical acumen.”	9
Practice-based learning and Improvement (PBLI)	Investigating and evaluating the needs of the population	“Firstly, I think the lesson on rationalising what we do is important. When thinking of strategies to help our patients in the future, whatever we advise must make sense. Like the health post in Bung, a lot of good can be rendered useless if we don’t rationalise what we do…” “Our surveys being conducted on healthcare accessibility and awareness feel more relevant and of greater importance as it will help us to understand the situation better and allow us to design programmes that will benefit the communities the most”	10
	Feedback to drive improvement	“…I realised that it is very important to receive feedback to gauge the effectiveness of what you are doing. Only after the first aid teaching, during the practical demonstration did I realise that many [local] students had poor grasps of English, yet during the first aid teaching, the translators overestimated the English-speaking ability of the students, leading to translation of only mainly the technical or complicated parts.” “I felt inspired that the hospital management team in Birat Eye Hospital were so receptive to feedback to improve the lives of the patients. Likewise, I’ll adopt a spirit of self-improvement”	8
Interpersonal and Communication Skills (ICS)	Use of non-verbal cues	“Working through translators, good communication skills become even more essential. I managed to apply the skills that the school taught us when working through translators, such as how we should still try to directly engage with the patient through things such as body language, hand gestures or facial expressions. I learnt a lot about how to make patients feel engaged and connected to you even though I was speaking through a translator.” “….I am the first contact with the patient and a lack of professionalism on my part will diminish their trust in our health screening. This included things like being nonjudgmental when villagers reported histories like they started smoking 4–5 cigarettes from 12…”	8
	Communicating across language barriers	“I also learnt the value of learning another’s language in building trust - the action of putting in effort to learn simple phrases creates a comfortable and warm atmosphere in a foreign place.” “One of my goals over the next 1–2 years is to bring it up to a level whereby I can conduct a medical consultation in their native language. This is especially important as many senior citizens in Singapore are not able to converse in English; if concepts are boiled down to first principles, they can be communicated even through cultural and language barriers.”	9
Systems-based Practice (SBP)	Healthcare systems	“I learnt how a hospital could be run efficiently and effectively, despite less resources (compared to Hospitals in Singapore for example)” “This experience in understanding the bigger picture of the healthcare system will help me along the way as it I will begin to consider these factors in the management of patients in the future.”	11
	Healthcare delivery	“I learnt about the healthcare delivery chain, which starts from transport to the hospital all the way to acute care in the hospital and finally rehabilitative care in the community setting. If there is a break at any point of the chain, it compromises the effectiveness of the healthcare system.”	10
	Resourcefulness & adaptability	“Improvising & using the bottom of the mineral water bottle for collecting the urine for urinalysis” “We learnt to be practical in resource management, using the structures and resources available to conduct screening camps.”	9
	Health equity	“Patients should be treated equally, without bias, no matter their racial, financial, ethnic background, among others. Healthcare is not reserved for any specific class of citizens.” “I will remember that not everyone is as privileged as Singaporeans are to have such an accessible healthcare system and easily accessible basic health knowledge (+ education in schools). I will also try to explore the fields of public health and health education to learn more about what can be done in less developed countries.”	10
	Health accessibility	“I learnt that healthcare accessibility is a major problem in countries such as Nepal. The most obvious visible reason was income inequality. Most of the villagers lived in simple houses made of hay and worked in the agricultural sector. Hence, they do not have the financial means to afford a cataract surgery or pay for travel to the Hospitals. “The health post in Bung is not frequented and used maximally since it is located high up the mountains and it is inconvenient for the people, especially the sick, to travel there.”	11
Professionalism (P)	Ethics	“In discussions, the team discussed how maintaining proper surgical conditions were important. Although we were providing the services free of charge and providing it in a different country, we should still try to replicate the same standards that we uphold in Singapore. “I learnt about the importance of having a good local partner to ensure success of the project when doing community work in a foreign place as without a good local partner, our project would not be sustainable as no work will be done when we are away from the country.”	8
	Role modelling	“I believe that the Dr A and Dr B have set a very good example in showing that healthcare is about the heart, and that we should always reach out to those in need of greater help.” “It was very heartwarming as Dr A decided then and there to help provide free eye screening and treatment to all the students from the school. “	7
	Teamwork & Leadership skills	“When working with a team on a project, I will Ensure that everyone is on the same page first before anything else. Failure to do so will increase the chances that the work we do will be less effective and will also increase the chances of miscommunications and misunderstandings. “Setting the direction for the project and discussing with team members to ensure that everyone’s goals are aligned”	7
	Interprofessional skills	“I think it is great that we have a sonographer onboard as she lets us investigate complaints which we would otherwise be unable to investigate beyond a superficial examination” “Saw teamwork between doctor and healthcare workers in Sotang to give best care to patients”	8
	Resilience	“.chatting with Dr K where he shared about the emotional resilience needed as doctors. Listening to each patient with an open heart and mind is not something easy and is emotionally exhausting. While I am not sure how we can build on this emotional depth, one important takeaway from that is to find our source of emotional avenue where we can express our emotions and recovering. It was also a good reminder to not shunt our emotions as an unhealthy defense mechanism.” “Seeing how the people cope despite the limitations of the healthcare system and infrastructure has given me a better insight into what it means to be resilient and to cope with difficult situations”	7

#### Patient care (PC)

Their reflections depicted the experience of *humanism*. Besides attending to the patients, the students observed how the doctors, translators and physiotherapists interacted with the patients. This allowed them to appreciate different facets of patient care such as understanding patients’ unique concerns besides the medical complaints and seeing them as an individual rather than a collection of symptoms and signs. *Socioeconomic and cultural determinants of health* were another two emerging themes of this domain. As one student reflected, “an elderly couple with social issues that greatly outweighed their medical ones, leading me to rethink how to we treat patients”. Seeing them in-situ within their villages and communities allowed the students to appreciate how their lifestyle, habitat and beliefs could influence their presenting medical complaints and health behaviors. For example, students noticed that despite medical advances and awareness, villagers preferred to follow the practice of being isolated during the menstrual cycle or deliver at home instead of using a birthing center due to their own cultural beliefs.

#### Medical knowledge (MK)

Besides clerking for the patients, the students took on the roles of a pharmacist and a triage nurse which helped them improve the understanding of the patient’s healthcare journey and narratives. Students also worked closely with the doctors who would supervise all the cases they saw. This opportunity allowed them to “use medical knowledge to correlate the clinical presentation with the disease” and *apply their medical knowledge* in a safe, protected environment.

#### Practice based learning and improvement (PBLI)

Interactions with the stakeholders especially enabled the students to appreciate the role of PBLI in striving for quality care for the villagers. As this OCIP doesn’t involve any NGOs, the students had the chance to directly interact with the village leaders and clinic leads to understand the healthcare issues in the village and brainstorm on solutions. Through conversations with these stakeholders, they were able to “understand the situation better and design programmes that will benefit the communities the most.” In this process, they learnt how best to *investigate and evaluate the needs of the population* and the importance of *regular feedback to improve the system*.

#### Interpersonal and communication skills

During the OCIP, the patients mainly spoke the Nepali language which indeed created a challenging *language barrier*. When the students had to work around this barrier, it allowed them to appreciate the importance of *non-verbal communication* as well as accuracy in understanding the patients’ narrative when taking a history from them. As one student described, students learned “how to make patients feel engaged and connected to you even though I was speaking through a translator.” During the daily reflections sharing, the physicians shared their communication challenges back in multiracial Singapore where knowing English alone is insufficient as each of the elderly patients speak their ethnic dialect. This reflection allowed the students to relate the experience to the situation In Singapore and reflect on how they would *communicate across language barriers*.

#### Systems based practice (SBP)

This OCIP was designed in a way that the team has to trek through the mountains from the nearest town to reach the villages for medical service provision and training. This follows the villagers’ journey should they need to travel to a tertiary hospital as the roads are not conducive for vehicular travel. The experience made the students realize how such a system can especially impact the speed of treatment in times of emergencies. During reflections, the physicians also shared that although Singapore is a developed country, for an elderly or disabled patient, their frequents trips to the hospital for multiple medical appointments is comparable. Hence, an ideal situation may be to have a strong primary healthcare facility near their homes, staffed by health professionals who have built a good rapport with the villagers and can manage common chronic conditions. It was commonly reflected that such experiences and sharing enabled them to *understand healthcare delivery* in low resource settings and relate it back to practice at home. Many reflected on *health inequity* as they saw how those living in the mountains were disadvantaged due to *inaccessibility* by virtue of the terrain or when they were unable to afford transport via helicopter to reach a tertiary hospital when time critical care is needed. In addition, the health post at these mountainous villages were often left unattended unlike those along more popular trekking routes like the Everest Base Camp trek or in the city. This created an unreliable system and affected the confidence the villagers have on the healthcare providers. Birthing centres were also present, but they were located on the top of a hill which was challenging for pregnant ladies to travel to. Hence people defaulted antenatal follow-ups and delivered at home. Such experiences brought about reflections on *healthcare systems, accessibility and delivery. Specifically, students highlighted the importance of “understanding the bigger picture of the healthcare system in the management of patients.”*

#### Professionalism

Many themes emerged under the domain of professionalism, such as the *ethics* around such short-term mission trips as well as *role modelling* when the students saw how the local doctors worked hard for the underprivileged population. Students reflected that healthcare is all “about heart” and they should “always reach out to those in need of greater help.” Experiencing healthcare in a low-income setting also brought about a *sense of gratitude*. Concurrently, organizing and conducting the trip together with different healthcare professionals provided the platform for the development of *teamwork, leadership and interprofessional skills*. Lastly, through their experience and reflections, the students reflected on their *self-resilience* as well as the resilience of the Nepalese people in managing with the minimum. Students observed that witnessing how Nepalese people navigate challenges despite limitations in healthcare infrastructure provided them with “a better insight into what it means to be resilient and how to cope with difficult situations.”

**Fig. 1:**
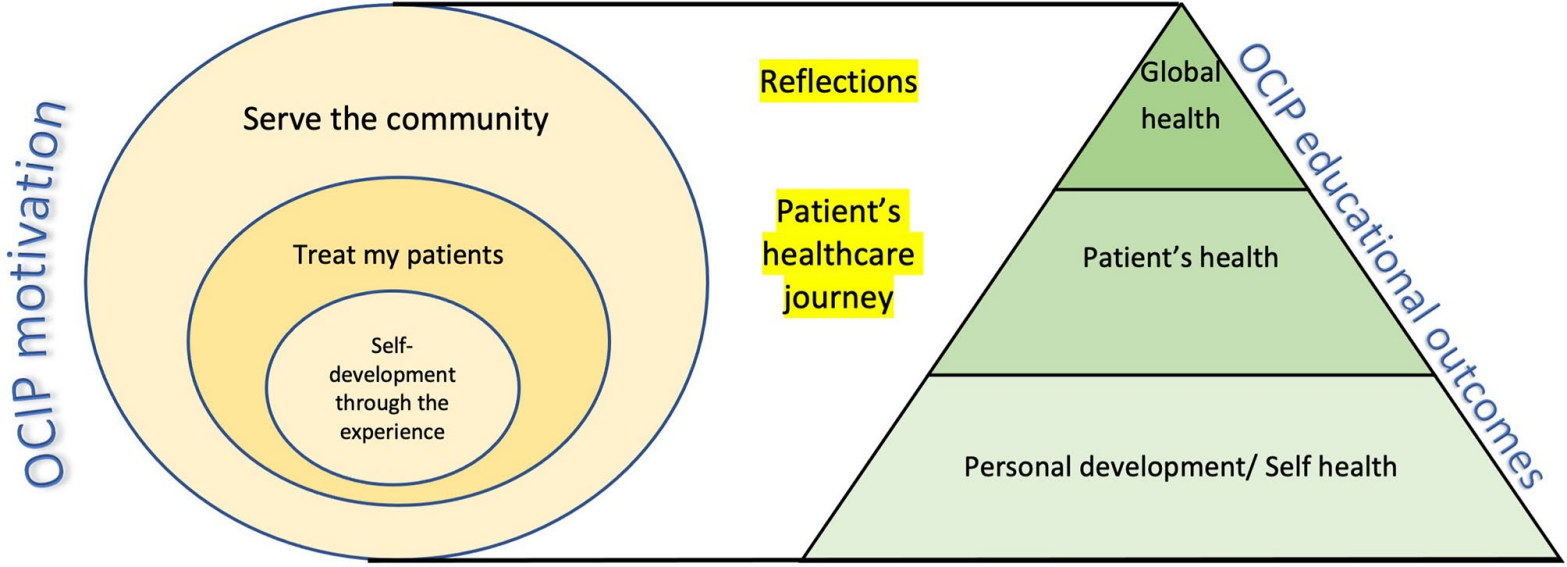
Framework for OCIP learning outcomes

The ACGME framework is broad enough to encompass the various themes from the students’ reflections. Interestingly, these themes refer to the soft or non-technical skills (NTS) in the medical curriculum. These themes also fall within the domains of global health education (socioeconomic and cultural determinants of health, PBLI, SBP), personal (teamwork & leadership skills, resilience) and professional (humanism, MK, ICS, interprofessional skills) development. Teaching the NTS is challenging and may sometimes be perceived as less important by the students. Hence, we propose an alternative conceptual model (Fig. 1) to highlight learning outcomes from OCIPs. It aims to help the facilitator and learner in reflecting on their experiences, converting them into learning moments and effectively consolidating learning outcomes in an OCIP. Our framework takes the form of a pyramid, with “Personal Development” forming its base, “Professional Development” building upon that foundation and ultimately capped with “Global Health Awareness”. It is structured as such because it is imperative for the learner to develop personal competencies and attributes to be in a comfortable zone, to glean the higher-order professional and global health skills offered by an OCIP experience. For example, without addressing personal competencies such as teamwork or adaptability to the challenging environment, students may struggle to progress to the next stage of learning professional competencies. Only by adequately addressing these two foundational skills, can students develop a deeper appreciation for global health principles, such as social determinants of health. Understanding the students’ motivations pre-trip can set the learners’ agendas and shape the experiential learning outcomes. Lastly, reflections during the trip and a healthcare journey approach can meaningfully contribute to reaching these outcomes.

## Discussion

This study sought to understand Singaporean medical students experience and learning outcomes of the OCIP. While findings from this study echo the benefits of global health experience published elsewhere [8, 10, 16], this is the first study to show how the OCIP experience could translate to various facets of ACGME domains. Our results demonstrate that OCIP is relevant to undergraduate medical education and could be a pedagogical tool for acquiring ACGME competencies as well as skills relevant to their personal, professional development and global health understanding.

The OCIP provides the opportunity to utilize both experiential learning [17, 18] and reflections, which are powerful pedagogical tools in medical education and part of the Kolb’s learning cycle. It provides the space to experience medicine in a more relaxed setting. The dedicated sharing time allows them to reflect and conceptualise the experience and eventually test out what they have learnt the following day [17]. The experience, reflection, abstract conceptualisation and experimentation are all part of the Kolb’s cycle.

The OCIP also contributes to the transformative learning process [19]. The students had certain assumptions at the start of the OCIP, which were challenged during the trip. Some of the self and group reflections evoked deep discussions which brought about a change in their perspectives. This is similar to studies which show that critical reflection of experiences serve as a pedagogical approach to learn complex concepts [20, 21]. For example, a successful physician is seen as one who can diagnose a patient’s problem and prescribe the appropriate management. However, in the low-resource setting, there was the realization that such skill would not suffice in the optimal long-term management, due to the scarcity of treatment or the inability of villagers to travel regularly to tertiary hospital for continued treatment. Thus, a “health systems” thinking process would be required to address the patient’s problems. Upstream problems (e.g., sanitation, diet) need to be addressed and active effort needs to be made for effective health education and preventative health. Allied healthcare may need to be stationed at the village health posts. The physician should be able to effectively communicate a diagnosis to the villagers and help them understand the impact of illness and treatment noncompliance on their lives such that they follow up on their treatment. And most importantly there should be a system to ensure continuity of care after the departure of overseas physicians. From this experience, it became evident that a successful physician should possess strong leadership skills and ability to bring all of these together.

Such an experience showed the students that a successful physician also needs to have NTS. The Lancet Commissions have proposed a new approach in medical education that focuses on teaching NTS to address health inequity [22]. These topics are also important to develop a future generation of doctors who are community and socially responsible [20, 23]. However, these are challenging topics to teach. The OCIP experience generated the importance of NTS - such as PC, ICS, SBP and professionalism. A well designed OCIP can facilitate the learning of these challenging concepts [24]. 

Findings from this study can pave the way for adoption of more relevant competencies to measure the impact of an OCIP. For example, cultural competence or humility has been one of the commonly used learning outcomes. However, the limitations of using this term as a learning outcome are being recognised, as it has not succeeded in reducing health disparities. In response, some have proposed a transnational [23, 24] approach to medical education and a global health curriculum to complement OCIPs. The transnational approach comprises both of medical and social competencies that allow the physician to manage patients in various settings. Some of the learning themes identified in this study fall within the transnational framework [24] and hence, these outcomes (e.g., health systems understanding) may be used to measure the educational effectiveness of an OCIP. Adopting a transnational approach may potentially result in incorporating new competencies into medical education to cultivate socially responsible physicians.

Our findings underscore the need to develop a curriculum for physicians leading OCIPs on how to facilitate the experiential learning through reflections [20]. A curriculum covering topics relevant to the practical and medical education aspects of an OCIP is much needed [4]. Although there are existing guidelines on global health ethics [2], infectious diseases, tropical and travel medicine, currently, there is no guideline on how to facilitate the experiential learning process of medical students during an OCIP. Our findings serve to act as an impetus to develop a more structured approach to OCIPs to ensure that its educational benefits are appropriately assessed.

This study has a few limitations. The study was based on a single OCIP group in Singapore which may limit the transferability of the findings. The physician leads of the OCIP group utilized reflections to facilitate learnings from the OCIP experience and hence there is uncertainty if similar learning outcomes will be achieved if an OCIP didn’t consist of reflective practice. This study explored the OCIP’s benefits solely from the perspectives of the medical student volunteers, leaving the viewpoints of local translators or local population unaccounted for. Further research work is warranted to include the perspectives of the community receiving help [25] to understand the OCIP’s experiential learning in a more holistic manner.

## Conclusion

The rich experience of an OCIP can provide valuable lessons that classroom or bedside teaching may not achieve. In today’s globalized world, as patient care becomes more complex, it is essential to be an all-rounded physician. The experiential learning from OCIPs can facilitate this development. Future steps should focus on how to make such trips more impactful and relevant for the community it serves and to develop a pre-trip checklist of competencies that encompasses the essential NTS required for such trips.

(3897 words)

### Electronic supplementary material

Below is the link to the electronic supplementary material.

Supplementary Material 1

## Data Availability

All relevant data are within the manuscript.
