# Supplementary material for: Medical students’ experience and learning outcomes of overseas community involvement project: a qualitative study
Source: BMC Med Educ. 2024 Aug 8;24:854. doi: 10.1186/s12909-024-05560-6 (PMC11311946; doi:10.1186/s12909-024-05560-6)
Supplement: Supplementary file 1 — Supplementary Material 1 [file 12909_2024_5560_MOESM1_ESM.docx]

Supplementary materials

Definition of ACGME core competencies

| Patient Care (PC) | PC refers to obtaining accurate information from the patient and with the knowledge and skills, provide a patient centered management appropriate in his/her context. This also refers to them practicing within the scope of their abilities with the correct judgement. |
| --- | --- |
| Medical Knowledge (MK) | MK refers to having sound basic science and clinical knowledge with the ability to use the knowledge and apply good analytical and problem-solving techniques to treat the patient. |
| Practice Based Learning and Improvement (PBLI) | PBLI refers to the ability to assimilate and apply the medical and scientific knowledge that is evolving. At the same time, keeping one updated on new evidence and continuously investigating and evaluating patient care practices to improve the practice of medicine |
| Interpersonal and Communication Skills (ICS) | This refers to both the verbal and non-verbal language that is used when communicating with the patients, their relatives and to the healthcare colleagues they work with during the care of the patient. |
| Systems Based Practice (SBP) | This refers to the ability to incorporate cost awareness and risk/benefit analysis in patient care, working towards patient safety, advocating for quality and optimal patient care systems and coordinating care with other healthcare providers. |
| Professionalism | Professionalism refers to the attitude and behavior of the doctor towards their patients and colleagues. It refers to how they carry themselves, and are accountable to their patients, the health system and society. It also comprises of altruism, humanism and personal and professional growth and development. |
